# Developing quality measures for non-pharmacological prevention and rehabilitation in primary health care for chronic conditions: a consensus study

**DOI:** 10.1093/intqhc/mzad097

**Published:** 2023-12-07

**Authors:** Marie Louise Svendsen, Tina Veje Andersen, Hanne Soendergaard

**Affiliations:** DEFACTUM, Central Denmark Region, Olof Palmes Allé 15, 8200 Aarhus N, Denmark; DEFACTUM, Central Denmark Region, Olof Palmes Allé 15, 8200 Aarhus N, Denmark; DEFACTUM, Central Denmark Region, Olof Palmes Allé 15, 8200 Aarhus N, Denmark

**Keywords:** health care quality, quality measures, chronic disease, rehabilitation, secondary prevention, primary health care

## Abstract

Poor health-related behaviours are root causes of a large number of chronic conditions; however, this study is the first to develop guideline-based quality measures targeting health-related behaviours through generic non-pharmacological secondary prevention and rehabilitation in municipal primary health care for persons with chronic conditions.

From January 2020 to September 2021, a consensus study was conducted in accordance with the current scientific recommendations for developing guideline-based quality measures. A clinical expert panel (*n* = 11) was established and included a patient representative, health care professionals, researchers, and key specialists. The process for developing quality measures was led by methodologists and encompassed a modified Research and Development/University of California at Los Angeles (RAND/UCLA) study to evaluate consensus in the expert panel. The consensus recommendations were directed to a steering group including the Danish Ministry of Health, the Danish Regions, and the Local Government Denmark.

The expert panel rated 102 clinical practice recommendations. Consensus was demonstrated on 13 quality measures assessing whether the patients are offered participation in and adhere to: self-management, smoking cessation, physical exercise training, nutritional efforts, and preventive consultation on excessive alcohol consumption; whether the patients participate in a closing meeting, whether they are offered follow-up, and whether reasons for dropout are documented.

The identified quality measures constitute a framework for assessing the quality of non-pharmacological prevention and rehabilitation in municipal primary health care for persons with chronic conditions. The next steps focus on field testing of the quality measures to refine measure criteria and assess implementation. A close link between clinical practice, the evidence and practice recommendations, the data infrastructure, economic considerations, and national priorities was a key to the consensus process.

## Introduction

Chronic conditions often transcend disease groups [1], and the estimated prevalence of persons with two or more chronic conditions ranges from 59.6% by the age group 55–64 years to 87.6% by the age group ≥75 years [2]. Coexisting chronic conditions is significantly associated with poor health [3–5] and high utilization of health care services [6], and generic care pathways addressing multiple conditions are strongly needed alongside treatment for single chronic conditions [7, 8]. As poor health-related behaviours are root causes of a large number of chronic conditions [8], a key approach is to promote healthy lifestyle [3, 8–10]. Therefore, public health recommendations for physical activity, smoking, diet, and alcohol consumption [3] as well as clinical guidelines for single [11] and coexisting chronic conditions [7] direct towards healthy lifestyle. Self-management support is also considered a key component of care for persons with single [9, 10] and coexisting [12, 13] chronic conditions in order to empower the persons to manage their conditions more effectively, seek appropriate health care and live healthier lives. Persons with chronic conditions frequently receive care in primary health care [7, 14], and in most Nordic countries, the municipalities are responsible for disease prevention and rehabilitation [14]. However, in this setting no quality measures (QM) exist for generic care pathways targeting health-related behaviours in persons with single or coexisting chronic conditions [13].

In 2019, The Local Government Denmark initiated a programme on behalf of the Danish Government with the aim of establishing a framework for nationwide quality assessment of non-pharmacological secondary prevention and rehabilitation in municipal primary health care for persons with chronic conditions. This included the development of generic QMs addressed in this consensus study. The clinical topic of the QMs was selected and operationalized a priori to the consensus study by a dedicated expert panel (Supplementary Material, Fig. S1). Accordingly, the generic care pathway encompassed the main care components in municipal primary health care from an introductory needs assessment meeting until a closing rehabilitation meeting focusing on the areas of self-management, smoking habits, physical exercise, nutrition, and alcohol habits. Other important areas were allowed if they were identified as clinical significant during the consensus study. Furthermore, the target population was defined as all persons with chronic conditions participating in an introductory needs assessment meeting defined by the clinical practice recommendations from the Danish Health Authority [15].

### Aim

The objective of this study was to develop QMs for generic non-pharmacological secondary prevention and rehabilitation for persons with chronic conditions in primary health care (provided by the municipalities).

## Methods

A consensus study was conducted from January 2020 to September 2021. Dedicated methodologists and researchers (M.L.S., T.V.A., H.S.) led the study using the methodological framework demonstrated in the Supplementary Material, Fig. S1. Collaboration and strategic partnerships were established via the programme organization and a designated clinical expert panel in order to facilitate the QM development and implementation [16] (Supplementary Material, Fig. S2). A consensus study in the expert panel established the recommendations on the QMs. The consensus study included three formal meetings and two online surveys (Table 1). The identified QMs were directed to a steering committee including the Danish Ministry of Health, Local Government Denmark, the Danish Health Data Authority, the municipalities, and representatives for the IT-infrastructure in the municipalities (Supplementary Material, Fig. S2). The directed QMs were supplemented by an outline of the expert panel members’ comments.

**Table 1.** Process: the expert panel.

The consensus study, steps	Objective	Panellists, *n* (%)
Meeting 1	Introduction to the methodological framework for developing quality measures	9 (81.8)
Online: expert opinions	The clinical expert panel reviewed the list of recommendations and was allowed to supply to the list if clinical important areas were missing (online, anonymous)	10 (90.0)
Online: Survey 1	Rating the full list of recommendations (online, anonymous)	11 (100)
Meeting 2	Evaluating consensus: quality measures^a^	10 (90.9)
At-meeting Survey 2	At-meeting mail voting on collectively recommending the 13 prioritised quality measures (anonymous)^a^	9 (81.8)
Meeting 3	Closing remarks from the expert panel members on the quality measures and the future process of practice testing and implementation	9 (81.8)

a One panel member was unable to participate in the second part of meeting 2 and the at-meeting survey.

This study follows the most recent scientific recommendations for developing guideline-based QMs [16–19], and follows the Standards for QUality Improvement Reporting Excellence (SQUIRE) 2.0 guidelines [20].

### Clinical expert panel formation

The interdisciplinary clinical expert panel of 11 members included frontline staff and leaders of primary health care rehabilitation centres (*n* = 5), a patient representative (*n* = 1), key researchers in the area of chronic conditions, cross-sectoral care, and rehabilitation of whom one represented the hospitals (*n* = 2), and key experts representing the Danish Health Authority’s official clinical practice recommendations for chronic conditions (*n* = 1), the national infrastructure of the approximately 80 official clinical quality databases (*n* = 1), and the IT-infrastructure in primary health care (*n* = 1). A representative of general practice declined to participate. The expert panel was assembled based on the following criteria of representation: relevant reflection of disciplines, sectors and geographical areas, the patient perspective, and key clinical and quality infrastructure specialists.

### Guidelines and clinical practice recommendations

The official clinical practice recommendations from the Danish Health Authority constituted the evidence-base for the consensus study because they specifically address the municipal setting [21–24]. Evidence and clinical guidelines for generic care pathways for single and coexisting chronic conditions are generally scarce and in particular for the municipal setting [12, 13, 21]. The official Danish recommendations are in agreement with Western clinical guidelines for single chronic conditions [11] and the few existing guidelines for coexisting chronic conditions [7].

Thus, included in this study were recommendations on non-pharmacological secondary prevention and rehabilitation in municipal primary health care for persons with chronic conditions [21] alongside selected single disease clinical practice guidelines focusing on rehabilitation in primary health care for cardiac disease [22], chronic obstructive pulmonary disease [23], and diabetes mellitus [24]. To specifically address coexisting chronic conditions, a Cochrane review on interventions for improving outcomes in patients with multimorbidity in primary care and community settings was included [4]. The recommendations on prevention and rehabilitation in municipal primary health care are based on the highest level of evidence and are generic for persons with chronic conditions [21]. The working group behind the recommendations involved the main national scientific societies operating in the fields of secondary prevention and rehabilitation in primary health care, and included the Danish College of General Practitioners, the Danish Society of Public Health, The Danish Society for Physiotherapy, Danish Societies for Nursing, Danish Societies for Occupational Therapy, the Danish Society for Clinical Nutrition, and a patient organisation [21].

### Consensus study on selecting quality measures

All clinical practice recommendations pertinent to the municipal setting were extracted from the reviewed clinical recommendations and guidelines [4, 21–24]. The expert panel reviewed the list of recommendations and was allowed to supply to the list if clinical important areas were missing. Next followed a modified Research and Development/University of California at Los Angeles (RAND/UCLA) study [25]. In an anonymous online survey, the expert panel rated the full list of clinical practice recommendations on a scale from 0 to 10 (Survey 1). The source of each clinical recommendation was stated; also when supplied by members of the expert panel. Rating criteria was the importance of each clinical recommendation to fulfil the aim of the quality assessment (0 = least important, 10 = most important). On a formal meeting, the collected results from Survey 1 were fed back to the expert panel as prioritized QMs and the panel members had the opportunity to discuss their ratings in light of the results. The responsible methodologists prioritized the QMs based on the following criteria: first, median ratings above 7 and secondly, suitable for assessing health care performance on the main generic chronic care components recommended by the Danish Health Authority (self-management, physical exercise, nutrition, smoking, alcohol) [21] (Supplementary Material, Table S1). Based on feedback from the expert panel, the second criterion was selected in order to elucidate that joint decisions were made in the clinical setting about each care component. Thereafter, an anonymous mail voting was performed at the meeting, and each panel member rated the extent to which they agreed to collectively recommend 13 prioritized QMs on a 0–10 scale (0 = totally disagree, 10 = totally agree) (Survey 2).

### Analysis

Proportions, medians, and ranges were analysed using descriptive statistics. Consensus agreement on the 13 QMs (Survey 2) were analysed by a common classification used: ‘No consensus’ (median score 0–3), ‘Uncertain’ (median score 4–6), and ‘Consensus’ (median score 7–10) [25]. Consensus recommendations based on median score 7–10 were subjected to veto approval by the expert panel to account for potential disagreements if all participant ratings did not fall within the 3-point range [25].

## Results

Between 9 (81.8%) and 11 (100%) of the panel members participated in three formal meetings, the online review of the official clinical practice recommendations, and two surveys (Table 1). The expert panel supplied the list of 50 official clinical practice recommendations with further 52 specific clinical areas in the defined care pathway. The provided areas were mainly refining the care components reflected in the recommendations; for example, offering a specific care component was supplied with adherence to and dropout from that component. Results of the anonymous voting (Survey 1) are illustrated in Table 2 for the clinical guidelines and recommendations that establish the prioritized QMs. Supplementary Material, Table S1 displays the results for the full list of 102 clinical practice recommendations. Voting (Survey 2) on the agreement on collectively recommending the 13 prioritized QMs demonstrated consensus in the expert panel (median 9, range 5–10) and was followed by veto approval by the panel members. The 13 prioritized QMs are shown in Table 2.

**Table 2.** Results from survey 1 (*n* = 11): the identified 13 quality measures and median ratings of the recommendations behind.

Quality measures no. 1–13	Clinical practice recommendations and guidelines	Ratings, median (range)
1: Proportion of persons who are offered participation in self-management	Offering self-management	10 (5, 10)
2: Proportion of persons who are offered smoking cessation	Offering smoking cessation	8.5 (5, 10)
3: Proportion of persons who are offered physical exercise training	Offering physical exercise training	10 (8, 10)
4: Proportion of persons who are offered nutritional efforts	Offering nutritional efforts	10 (5, 10)
5: Proportion of persons who are offered preventive consultation on alcohol consumption	Offering preventive consultation on alcohol consumption	8 (5, 10)
6: Proportion of persons who participate in a closing meeting	Closing rehabilitation meeting	10 (8–10)
7: Proportion of persons where reasons for dropout are documented		
	Reasons for dropout (finalizing the care pathway)	9 (0–10)
	Reasons for dropout from self-management	6 (0, 10)
	Reasons for dropout from smoking cessation	8 (0, 10)
	Reasons for dropout from physical exercise training	7 (0, 10)
	Reasons for dropout from nutritional effort	8 (0, 10)
	Reasons for dropout from preventive consultation on alcohol consumption	8 (0, 10)
8: Proportion of persons who are offered follow-up at the closing meeting		
	Follow-up on the person’s activity and adherence	10 (5, 10)
	Follow-up on goal setting	9 (0, 10)
	Follow-up in a group or individually	5 (0, 10)
	Maintenance and follow-up on physical exercise training	8 (5, 10)
9: Proportion of persons who adhere to self-management	Actual participation in chronic disease self-management	8 (0, 10)
10: Proportion of persons who adhere to smoking cessation	Actual participation in smoking cessation	8 (0, 10)
11: Proportion of persons who adhere to physical exercise training	Actual participation in physical exercise training	8 (0, 9)
12: Proportion of persons who adhere to nutritional efforts	Actual participation in nutritional efforts	8 (0, 10)
13: Proportion of persons who adhere to preventive consultation on alcohol consumption	Actual participation in preventive consultation on alcohol consumption	7.5 (0, 10)

### Panel members’ comments

The panel members disputed the lack of high-quality evidence on generic care pathways for chronic conditions; for example, translating the QMs for adherence to the care components into measurable fractions with clearly defined numerators and denominators are challenged by the lack of evidence. Also, concerns were reported about the lack of infrastructure related to data and IT-systems as well as the burden of data collection in terms of time and costs. Integrating registrations in the existing electronic patient records was perceived essential in order to reduce the burden. Furthermore, the expert panel members stressed the importance of coherence between the quality assessment in the municipalities and the established infrastructure of the clinical quality databases (primary targeting the hospitals) in order to assess cross-sectoral care.

## Discussion

### Statement of principal findings

The identified set of QMs constitutes a framework for assessing the quality of non-pharmacological secondary prevention and rehabilitation in municipal primary health care for persons with chronic conditions targeting healthy lifestyle and self-management. Key to the consensus process was a close link between the clinical practice, existing clinical practice recommendations based on the highest level of evidence, and strategic partnerships to safeguard economy and coherence with the national IT- and quality infrastructure.

### Strengths and limitations

The study adheres to the most recent scientific recommendations for developing guideline-based QMs [16–19] and is reported in accordance with the SQUIRE 2.0 guidelines [20], thereby improving the validity and replicability of the study. This includes a strategic decision to develop the QMs with dedicated methodologists to lead the process, the establishment of a multidisciplinary expert panel with key clinicians and methodologists, the use of established consensus methodology, coherence between the clinical and the political/administrative sphere, and distinct transparency in study completion and reporting [16–19]. However, the validity of the chosen QMs to capture the most effective components of care may be questioned because of the limited evidence for generic care pathways for single [8] and coexisting [4] chronic conditions in municipal primary health care. As considered best practice in the development of guideline-based QMs particularly in case of weak evidence, this study applied expert opinions and consensus methods [19]. Thus, the selection, composition, and functioning of the expert panel are important considerations [17]. Key clinical and methodological stakeholders and a patient representative were included in the expert panel all of which are reported as facilitating factors for the process of developing and implementing guideline-based QMs [16]. Although no general agreement on the role of patients in the development of QMs exists [16], the patient representative safeguarded that the patient perspectives were actively debated and represented in the consensus process. Furthermore, the expert panel included key specialists in order to support an integrated approach between the QMs and the clinical practice recommendations, the established national quality infrastructure, and the IT-infrastructure. Integrating these elements is proposed to be prerequisite for successful guideline-based QMs [16, 19]. However, no general practitioners were represented in the expert panel possibly weakening the emphasis on the cross-sectoral pathway, including patterns of referral.

### Interpretation within the context of the wider literature

The results of this study promote an individualized multidisciplinary programme of interventions that support healthy lifestyle and self-management as well as scheduled follow-ups [21]. This is in agreement with the clinical guidelines for single chronic conditions [11] and the few existing clinical guidelines and practice guidance for the management of coexisting chronic conditions [7, 12, 13]. Thus, evidence supports the specific care components in the defined generic care pathway by demonstrating benefits of healthy behaviours to prevent, treat, and reverse the root causes of common chronic conditions [8]. This includes diet changes in primary and secondary preventions of chronic conditions [8, 11], and physical activity as a key behaviour related to the prevention and treatment of many chronic conditions [8, 11]. Furthermore, substance abuse and in particularly smoking remains the leading cause of preventable deaths [3, 8]. As consistently recommended for coexisting chronic conditions [7, 12, 13], the expert panel stressed the importance of patient preferences and shared decision making. However, lack of measurability is one of the main challenges in the development of QMs particularly related to individualized aspects of care such as shared decision making [16]. Suggested for implementation is a strategical decision to include shared decision making into quality improvement on the organizational or system level [16]. Correspondingly, the expert panel members agreed that the inclusion criteria for quality assessment is participation in an introductory needs assessment meeting defined by official clinical practice recommendations from the Danish Health Authority [15]; thus, the care pathway starts with an introductory needs assessment meeting based on standardized principles for the performance of the needs assessments, shared decision making, and the competences of the health care professionals [15].

The expert panel members raised concerns about the burden of data collection. This agrees with previous research demonstrating that difficulties in reaching consensus on guideline-based QMs predominantly concern feasibility and economical judgements [16]. Also, disjunction between the clinical and political sphere may hinder the development of QMs [16]. In this study, a parallel initiative aiming at developing a nationwide shared IT-infrastructure and data standardization between the primary health care centres will presumably facilitate the acceptance and implementation of the QMs. Furthermore, the collaborative organization of this study between the clinicians, the administrative level, and national priorities will most likely facilitate the acceptance and the implementation of the QMs [16].

### Implications for policy, practice, and research

The Global Burden of Disease study 2019 calls for rehabilitation to be brought close to communities as an integral part of primary health care [26], and the identified QMs are a first critical step to safeguard and improve the quality of care in the municipal setting for persons with chronic conditions. Future field testing and implementation will determine the usability of the QMs in practice and any burden of data collection [16, 17]. However, careful considerations must be taken before applying the identified QMs in other settings because the findings may be influenced by contextual factors due to the weak evidence and high confidence in expert opinions. Further evidence on generic care pathways for persons with chronic conditions in municipal primary health care is needed.

## Conclusions

The QMs constitute a framework for assessing and improving the quality of non-pharmacological secondary prevention and rehabilitation for persons with chronic conditions in municipal primary health care. The next steps focus on field testing of the QMs to refine measure criteria and assess implementation.

## Supplementary Material

mzad097_Supp

## Data Availability

The data underlying this article cannot be shared publicly because the expert panel members did not give written consent for their individual ratings to be shared (Surveys 1 and 2). Minutes of the meetings in the expert panel will be shared on reasonable request to the corresponding author.
