# Supplementary material for: Developing quality measures for non-pharmacological prevention and rehabilitation in primary health care for chronic conditions: a consensus study
Source: Int J Qual Health Care. 2023 Dec 7;35(4):mzad097. doi: 10.1093/intqhc/mzad097 (PMC10712901; doi:10.1093/intqhc/mzad097)
Supplement: mzad097_Supp [file mzad097_supp.zip › suppl_data/Suppl1_Figure1_unchanged.docx]

**SUPPLEMENTAL MATERIAL 1**

**Supplemental Figure 1. The methodological framework (adapted from ^1^)**


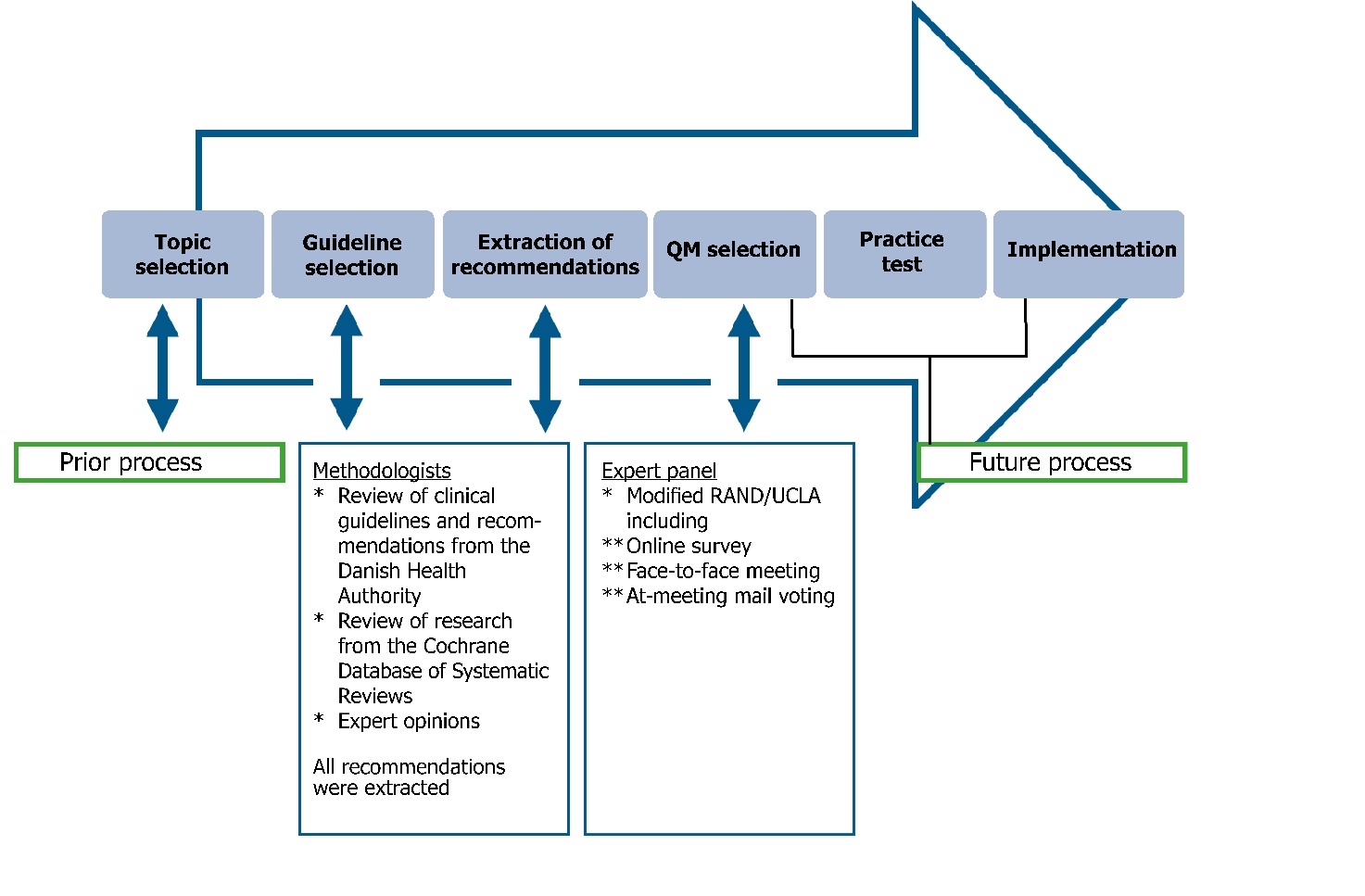


1. Kötter T, Blozik E, Scherer M. Methods for the guideline-based development of quality indicators--a systematic review. *Implement Sci* 2012; 7: 21. Figure 2, Overview of the process of guideline-based QI development, p. 5.
