# Supplementary material for: Developing quality measures for non-pharmacological prevention and rehabilitation in primary health care for chronic conditions: a consensus study
Source: Int J Qual Health Care. 2023 Dec 7;35(4):mzad097. doi: 10.1093/intqhc/mzad097 (PMC10712901; doi:10.1093/intqhc/mzad097)
Supplement: mzad097_Supp [file mzad097_supp.zip › suppl_data/Suppl2_Figure-2_ R_INTQHC-2023-03-0124_unchanged.docx]

## SUPPLEMENTAL MATERIAL 2

## Supplemental Figure 2. The organisational framework

* Proposals from the parallel initiative described in the article and from the expert panel; e.g., the legislative framework of the quality assessment, the IT-infrastructure, and the recommended quality measures.
