# Supplementary material for: Developing quality measures for non-pharmacological prevention and rehabilitation in primary health care for chronic conditions: a consensus study
Source: Int J Qual Health Care. 2023 Dec 7;35(4):mzad097. doi: 10.1093/intqhc/mzad097 (PMC10712901; doi:10.1093/intqhc/mzad097)
Supplement: mzad097_Supp [file mzad097_supp.zip › suppl_data/Suppl3_Table-1_R_INTQHC-2023-03-0124_unchanged.docx]

## **SUPPLEMENTAL MATERIAL 3**

**Supplemental Table 1. Full list of recommendations: evidence base and panel ratings**

| **QM^1^** | **Recommendation** | **Evi-dence^2^** | **Rating, n (%)** | **Rating, median (range)** |
| --- | --- | --- | --- | --- |
|  | **Introductory meeting and needs assessment (as a care component)** | 1 | 11 (100) | 10 (9, 10) |
|  | - Specific elements of the meeting; e.g., assessing the person's functioning, motivation and risk factors | 1 | 6 (55) | 9.5 (6, 10) |
|  | - Actual participation | 6 | 10 (91) | 9 (2, 10) |
|  | - Types of care components presented at the introductory meeting | 6 | 7 (64) | 9 (0, 10) |
|  | - Goal setting for the individual care components | 6 | 9 (82) | 9 (0, 10) |
|  | - Accepted care components | 6 | 8 (73) | 8.5 (6, 10) |
|  | - Individualised aspects of the meeting | 6 | 8 (73) | 8.5 (4, 10) |
|  | - Duration of the rehabilitation program | 4 | 9 (82) | 8 (6, 10) |
|  | - The motivation of the health professionals' as a barrier to participation | 6 | 7 (64) | 8 (0, 10) |
|  | - Reasons for rejecting the care components | 6 | 9 (82) | 8 (0, 10) |
|  | - Reasons for not attending the introductory meeting | 6 | 9 (82) | 8 (0, 10) |
|  | - Participation in individual or group care | 6 | 9 (82) | 8 (0, 10) |
|  | - Distance as a barrier to participation | 6 | 7 (64) | 7 (4, 8) |
|  | - The persons' motivation as a barrier to participation | 6 | 8 (73) | 7 (3, 10) |
|  | - Goal setting at the initial meeting | 6 | 9 (82) | 7 (0, 10) |
|  | - Date of the initial meeting; i.e.; time from hospital admission to start of rehabilitation | 4 | 8 (73) | 6.5 (0, 10) |
|  | - Dealing with barriers to participation | 2 | 8 (73) | 6.5 (0, 10) |
|  | - Restarting rehabilitation | 6 | 9 (82) | 6 (0, 8) |
|  | - Introductory meeting with caregivers | 6 | 7 (64) | 6 (1, 10) |
|  | **Finalizing the care pathway (as a care component)** | 1 | 11 (100) | 10 (9, 10) |
| 6 | - Closing rehabilitation meeting | 1 | 9 (82) | 10 (8, 10) |
| 8 | - Follow-up on the person's activity and adherence | 1 | 8 (73) | 10 (5, 10) |
|  | - Communication and coordination with general practitioners and others involved | 1 | 7 (64) | 9 (3, 10) |
| 8 | - Follow-up on goal setting | 6 | 9 (82) | 9 (0, 10) |
| 7 | - Reasons for dropout | 6 | 9 (82) | 9 (0, 10) |
|  | - Plan for the person's future adherence | 6 | 9 (82) | 8 (5, 10) |
|  | - Reasons for non-adherence | 6 | 9 (82) | 8 (0, 10) |
|  | - Shared evaluation | 6 | 9 (82) | 7 (5, 10) |
|  | - Closing rehabilitation meeting with relatives | 6 | 9 (82) | 7 (3, 10) |
| 8 | - Follow-up in a group or individually | 6 | 9 (82) | 5 (0, 10) |
|  | **Chronic disease self-management (as a care component)** | 1 | 11 (100) | 9 (6, 10) |
| 1 | - Offering self-management | 1 | 9 (82) | 10 (5, 10) |
|  | - Specific elements; e.g., risk factor information | 1 | 9 (82) | 8 (7, 10) |
|  | - Education in activities of daily living | 1 | 9 (82) | 8 (5, 10) |
|  | - Assessment of the effect of self-management | 6 | 9 (82) | 8 (3, 10) |
|  | - Psychosocial approach | 2 | 9 (82) | 8 (0, 10) |
| 9 | - Actual participation in self-management | 6 | 9 (82) | 8 (0, 10) |
|  | - Offering patient education | 2 | 9 (82) | 7 (0, 10) |
|  | - Involvement of caregivers | 4 | 9 (82) | 6 (3, 9) |
|  | - Organisation; e.g., format and duration of sessions | 1 | 9 (82) | 6 (2, 10) |
|  | - Self-management approach | 3 | 9 (82) | 6 (0, 10) |
| 7 | - Reasons for dropout from the self-management component | 6 | 9 (82) | 6 (0, 10) |
|  | - Goal setting for self-management | 6 | 9 (82) | 6 (0, 10) |
|  | - Mental health; e.g., guidance in strategies to improve well-being | 1 | 9 (82) | 5 (5, 10) |
|  | - Competencies of the health care professionals | 1 | 9 (82) | 5 (0, 10) |
|  | - Pedagogical approach | 6 | 9 (82) | 5 (0, 10) |
|  | Smoking cessation (as a care component) | 1 | 11 (100) | 9 (6, 10) |
|  | - Reporting to the national clinical database for smoking cessation | 6 | 9 (82) | 10 (0, 10) |
| 2 | - Offering smoking cessation | 1 | 8 (73) | 8.5 (5, 10) |
|  | - Smoking habits | 6 | 9 (82) | 8 (5, 10) |
| 10 | - Actual participation in smoking cessation | 6 | 9 (82) | 8 (0, 10) |
| 7 | - Reasons for dropout from smoking cessation | 6 | 9 (82) | 8 (0, 10) |
|  | - Specific elements of smoking cessation | 1 | 9 (82) | 8 (0, 10) |
|  | - Organisation; e.g., format and duration | 1 | 9 (82) | 8 (0, 10) |
|  | - Competencies of the health care professionals | 1 | 8 (73) | 5 (0, 9) |
| 3 | **Physical exercise training (as a care component)** | 1 | 11 (100) | 8 (5, 10) |
|  | - Offering physical exercise training | 1 | 8 (73) | 10 (8, 10) |
|  | - Supporting unsupervised physical exercise training | 1 | 9 (82) | 9 (5, 10) |
| 8 | - Maintenance and follow-up on physical exercise training | 6 | 9 (82) | 8 (5, 10) |
|  | - Test at the beginning and the end of physical exercise training | 6 | 9 (82) | 8 (0, 10) |
|  | - Organisation; e.g., format and duration | 1 | 9 (82) | 8 (0, 10) |
| 11 | - Actual participation in physical exercise training | 6 | 9 (82) | 8 (0, 9) |
|  | - Assessing unsupervised physical exercise training | 6 | 9 (82) | 7 (0, 10) |
| 7 | - Reasons for dropout from physical exercise training | 6 | 9 (82) | 7 (0, 10) |
|  | - Effect: increase in function or physical capacity | 6 | 9 (82) | 6 (4, 10) |
|  | - Competencies of the health care professionals | 1 | 9 (82) | 8 (0, 10) |
|  | - Type of supervised physical exercise training | 1 | 9 (82) | 8 (5, 10) |
|  | **Nutritional efforts (as a care component)** | 1 | 11 (100) | 8 (5, 10) |
| 4 | - Offering nutritional efforts | 1 | 9 (82) | 10 (5, 10) |
| 12 | - Actual participation in nutritional efforts | 6 | 10 (91) | 8 (0, 10) |
|  | - Maintenance of achieved changes | 6 | 9 (82) | 8 (0, 10) |
| 7 | - Reasons for dropout from nutritional effort | 6 | 10 (91) | 8 (0, 10) |
|  | - Nutrition assessment | 2 | 10 (91) | 7.5 (4, 10) |
|  | - Nutrition therapy | 1 | 10 (91) | 7.5 (0, 10) |
|  | - Competencies of the health care professionals | 1 | 10 (91) | 7.5 (0, 10) |
|  | - Nutrition counselling | 1 | 10 (91) | 7 (0, 10) |
|  | - Everyday cooking | 1 | 9 (82) | 6 (0, 10) |
|  | - Organisation; e.g., format and duration | 1 | 10 (91) | 6 (0, 10) |
|  | - Nutritional status at the beginning | 6 | 10 (91) | 5 (0, 10) |
|  | - Collaboration with others involved, e.g. dentists | 6 | 10 (91) | 5 (0, 8) |
|  | **Preventive consultation on alcohol consumption (as a care component)** | 1 | 11 (100) | 7 (5, 10) |
| 5 | - Offering preventive consultation on alcohol consumption | 1 | 9 (82) | 8 (5, 10) |
|  | - Maintenance of achieved changes | 6 | 9 (82) | 8 (5, 10) |
|  | - Specific elements of the consultation; e.g., counselling | 1 | 10 (91) | 8 (0, 10) |
| 7 | - Reasons for dropout from preventive consultation on alcohol consumption | 6 | 10 (91) | 8 (0, 10) |
| 13 | - Actual participation in preventive consultation on alcohol consumption | 6 | 10 (91) | 7.5 (0, 10) |
|  | - Outcome assessment on alcohol consumption | 6 | 10 (91) | 7.5 (0, 10) |
|  | - Organisation; e.g., format and duration | 1 | 10 (91) | 6.5 (0, 8) |
|  | - Alcohol consumption status | 6 | 10 (91) | 6 (0, 10) |
|  | - Competencies of the health care professionals | 1 | 10 (91) | 5.5 (0, 10) |
|  | **Other areas (as a care component)** | 6 | 9 (82) | 7 (0, 10) |
|  | **-** Coherent rehabilitation; e.g., coherence in the care pathway of rehabilitation and secondary prevention | 3 | 10 (91) | 8.5 (0, 10) |
|  | - Job retention | 2 | 10 (91) | 8 (0, 10) |
|  | - Mental health; e.g., assessment | 5 | 10 (91) | 7.5 (1, 10) |
|  | - Continued needs assessments | 1 | 10 (91) | 7 (2, 10) |
|  | - Screening for anxiety and depression | 2 | 10 (91) | 7 (0, 10) |
|  | - Telemedicine and rehabilitation | 3 | 9 (82) | 7 (0, 10) |
|  | - Co-morbidity | 6 | 9 (82) | 7 (0, 10) |
|  | - Physical parameters; e.g., BMI | 6 | 9 (82) | 6 (0, 10) |
|  | - Sociodemographic parameters; e.g., social network | 6 | 8 (73) | 6 (0, 10) |
|  | - Patient reported outcome measures | 6 | 10 (91) | 5 (3, 9) |
|  | - Contact with the employer | 6 | 9 (82) | 5 (0, 9) |

^1^ Quality Measure

^2^ 1=clinical practice recommendations on non-pharmacological secondary prevention and rehabilitation in municipal primary health care for persons with chronic conditions^1^, 2= clinical practice guidelines on rehabilitation for cardiac disease^2^, 3= clinical practice guidelines on rehabilitation for diabetes mellitus^3^, 4= clinical practice guidelines on rehabilitation for chronic obstructive pulmonary disease^4^, 5=Cochrane review on multimorbidity^5^, 6=expert opinions.

**REFERENCES**

1. The Danish Health Authority. Anbefalinger for forebyggelsestilbud til borgere med kronisk sygdom. Copenhagen: The Danish Health Authority; 2016.

2. The Danish Health Authority. National klinisk retningslinje for hjerterehabilitering. Copenhagen: The Danish Health Authority; 2013.

3. The Danish Health Authority. National klinisk retningslinje for udvalgte sundhedsfaglige indsatser til patienter med type 2 diabetes. Copenhagen: The Danish Health Authority; 2017.

4. The Danish Health Authority. National klinisk retningslinje for rehabilitering af patienter med KOL. Copenhagen: The Danish Health Authority; 2018.

5. Smith SM WE, O'Dowd T, Fortin M. Interventions for improving outcomes in patients with multimorbidity. Interventions for improving outcomes in patients with multimorbidity in primary care and community settings. *Cochrane Database Syst Rev* 2021:CD006560.pub3.
